# Novel Photoreactive Pressure-Sensitive Adhesives (PSA) Based on Acrylics Containing Additionable Photoinitiators

**DOI:** 10.3390/ma13225151

**Published:** 2020-11-16

**Authors:** Zbigniew Czech, Janina Kabatc, Marcin Bartkowiak, Adam Licbarski, Karolina Mozelewska, Dominika Kwiatkowska

**Affiliations:** 1 International Laboratory of Adhesives and Self-Adhesive Materials, Department of Chemical Organic Technology and Polymeric Materials, Faculty of Chemical Technology and Engineering, West Pomeranian University of Technology, Pułaskiego 10, 70-322 Szczecin, Poland; marcin.bartkowiak@zut.edu.pl (M.B.); adm_l@wp.pl (A.L.); karolina_mozelewska@zut.edu.pl (K.M.); 2 Department of Organic Chemistry, Faculty of Chemical Technology and Engineering, UTP University of Science and Technology, Seminaryjna 3, 85-326 Bydgoszcz, Poland; dominika.kwiatkowska@utp.edu.pl

**Keywords:** pressure-sensitive adhesive acrylics, photoreactive, additionable photoinitiators

## Abstract

A new class of additionable ultraviolet photoinitiators that can be used, through addition, for modification of the acrylic polymer chain and their influence of main properties of acrylic pressure-sensitive adhesives (PSAs) is described here. The photoinitiators studied are based on benzophenone, dibenzofuran and anthraquinone chromophores. The propyleneimine carbonyl is the reactive additionable group incorporated in the photoinitiator structure. First, the solvent-borne acrylic pressure-sensitive adhesive was synthesized and characterized. Then, a photoinitiator suitable for addition to the acrylic polymer chain possessing a carboxyl group was added before UV-irradiation. A mechanism of UV-initiated cross-linking reaction of acrylic PSA with additionable photoinitiators was done as well. The influence of the concentration and type of photoinitiator, UV-crosslinking time and UV-dose on peel adhesion, shear strength and tack of solvent-borne acrylic pressure-sensitive adhesives cross-linked by UV light was studied and presented here. It was found that the tack depends on the UV-dose and photoinitiator concentration. An increase of UV dose results in an increase of shear strength of acrylic pressure-sensitive adhesive (PSA) formulations.

## 1. Introduction

The phrase pressure-sensitive adhesive (PSA) means an adhesive composition that is characterized by the ability to adhere to a different surface by the simple application of light hand pressure. This composition remains permanently tacky [1]. These adhesives are widely used in single-sided, double-sided and self-adhesive labels, marking and labeling films, protective films, and in medical devices as gypsum and self-adhesive bioelectrodes [2,3,4,5]. Pressure-sensitive adhesives may be used as a waterborne, solvent-borne and as a solvent-free system. In the giant field of adhesives, the PSA make up, but a low percentage and the solvent-borne pressure-sensitive acrylic adhesives with about 240,000 tons per annum in Europe are almost a quantity negligible within this group. Other types of PSA in use nowadays include natural and synthetic rubber, silicones, polyesters, polyurethanes, ethylene-vinyl acetate copolymers (EVA) and polyether [6,7].

The number of possible applications of UV-cross-linkable materials has increased rapidly since the last decade. This is attributed to the productivity and environmental benefits that result from such technologies. The combination of the pattern with the UV-crosslinked PSA enables the production of films having zones of different adhesion–cohesion balance and offers new possibilities for the development of innovative tapes with new unique characteristics [8,9,10,11,12,13,14,15,16,17].

Generally, UV-crosslinked PSA systems operate at room temperature. Therefore, they are often used for the production of high-temperature coating systems, for example: calendered and hot-melt [18]. A survey of the field of UV radiation cross-linked PSAs includes 75 relevant and significant patents and 20 journal references. The science of UV-crosslinking has been transformed over two decades from a topic of esoteric research specialties into one of the major industrial technologies and is now a field of central importance in polymer science and technology. UV cross-linking technology, as opposed to thermal cross-linking, allows for rapid networking using high-intensity light sources and the use of heat-sensitive substrates.

This technology is based on the photoinitiation of radical and cationic reaction. The ultraviolet cross-linking process requires the addition of a suitable photoinitiator to the PSA system. The chemical structure and concentration of the photoinitiator strongly affect the cross-linking process.

In recent years, many studies have been carried out on the synthesis of new photoinitiators with well-defined and desired properties, for example, higher activity, slower migration, lower toxicity [19,20,21,22].

There are two types of photoinitiators that induce photocrosslinking. The first induces a free radical process in which low molecular weight polymers with photoreactive chains are converted during irradiation with UV light into highly cross-linked PSA acrylic films. The second are photoinitiators acting via an ionic mechanism (usually cationic) for ring-opening cross-linking of epoxy- and vinyl ether-based monomers and polymers [23,24,25,26,27].

The formation of free radical species through the absorption of UV light by the photoinitiator is necessary for the cross-linking process. There are known two groups of photoinitiators. Type I—these are initiators that decompose rapidly in their excited state, and as a result, the free radicals are formed. The type II photoinitiators act via hydrogen atom abstraction from the environment (monomer, solvent, etc.) to create a ketyl radical. An important factor is the photoreductive ability of the environment and is related to the carbon-hydrogen bond strength [28,29,30,31].

**Figure 1:**
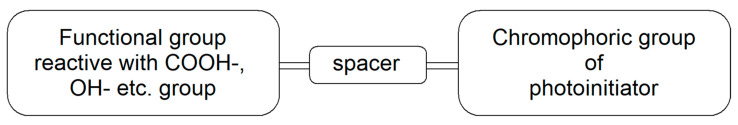
General scheme of saturated photoinitiators suitable for addition.

Additionable photoinitiators suitable for addition are a new class of typical radical photoinitiators [22]. They are not commercially available on the market. A characteristic feature of this type of photoinitiator is the presence of two different groups: chromophoric (e.g., benzophenone, anthraquinone or fluorenone) and conventional reactive chemical group (Figure 1).

Carboxyl groups undergo addition in the polymerization environment without causing a lack of byproducts and lead to high UV-cross-linked pressure-sensitive adhesives. Another approach to increase the cohesion of an acrylic PSA via UV cross-linking is the incorporation of defined additionable photoinitiators into a saturated polymeric chain.

Herein, we report the effect of novel suitable for addition photoinitiators on peel adhesion, tack and shear strength performance of synthesized acrylic pressure-sensitive adhesives (PSA) after UV cross-linking. The incorporation of novel additionable photoinitiators takes place after the polymerization.

## 2. Materials and Methods

### 2.1. Synthesis of Photoreactive Acrylic PSA

For the above-mentioned experiments, the model solvent-borne PSA was prepared using a 62 wt % of 2-ethylhexyl acrylate, 33 wt % of methyl acrylate and 5 wt % of acrylic acid mixture in ethyl acetate. For polymerization, 0.1 wt % thermal initiator azobisisobutyronitrile (AIBN) was used. The polymerization process was conducted under the following conditions: addition of the blend of monomers with AIBN into ethyl acetate before the polymerization 50 wt %, dosage time of residual monomers with residual AIBN 2 h, time of post-reaction 4 h. All monomers and AIBN are available from BASF (Ludwigshafen, Germany).

### 2.2. Amount of Solid Materials and Physical Data Synthesized Acrylic PSA

The amount of solid materials was determined, according to DIN EN 12092. The amount of unreacted monomer was determined using a gas chromatography system (GC Unicam 610 (ATI Unicam, Cambridge, UK)) equipped with integrator Unicam 4815, flame ionization detector (FID) and capillary column J&W DB-1.

Molecular weight determination was carried out with liquid chromatography method (LC) using a LaChrom-Merck system with Refractive Index (RI) Detector L-7490 and UV Detector L-7400 from Merck Hitachi (Darmstadt, Germany), equipped with a PLgel 106 Å column from Agilent Technologies, Inc. (Santa Clara, CA, USA)

### 2.3. Photoreactive Modification of Acrylic Pressure-Sensitive Adhesive

The acrylic PSA prepared was modified through the addition of additionable photoinitiators in concentration between 0 (PSA is not photoreactive) and 2 wt % according to polymer content. The transformation of the carboxyl group of a pressure-sensitive adhesive takes place after the polymerization by the addition reaction in the polymerization media without side products and leads to UV-cross-linkable highly efficient pressure-sensitive adhesives based on acrylics. Effective anchoring of additionable photoinitiators (photocrosslinkers) was analyzed using IR techniques. The photoinitiators being suitable for addition are presented in Table 1.

### 2.4. Coating of Photoreactive Modified Acrylic PSA and UV Crosslinking

Solvent-borne photoreactive acrylic PSA containing incorporated into polymer chain additionable photoinitiators were coated on 50 μm-thick polyester film. After coating, the resulting one-sided tapes with polyester film as carriers were dried for 10 min at 110 °C and the coat weight was 60 g/m^2^. The one-sided tapes were cross-linked through 60 s under UV-C lamp 400 T from Hönle company (Gräfelfing, Germany).

### 2.5. Determination of Tack, Peel Adhesion and Shear Strength of Crosslinked Acrylic PSA Layers

The following adhesion properties (tack, adhesion, cohesion and shrinkage) were determined by standard A.F.E.R.A. (Association des Fabricants Europeens de Rubans Auto-Adhesifs) procedures: AFERA 4015—tack, AFERA 4001—peel adhesion and AFERA 4012—shear strength. The test was conducted with a Zwick/Roell Z-25 testing machine (Zwick Roell, Wrocław, Poland).

### 2.6. Tack

Pressure-sensitive tack is the property of the adhesive layer related to bond formation. The method, according to AFERA 4015, is very simple and can be conducted using common tensile strength test machines. A sample of PSA-coated tape 1-in wide and 7-in long is bonded vertically to a clean test plate made of steel, at least 10 lineal cm in firm contact. The steel plate is clamped in the jaws of a tensile testing machine Zwick-Roell Z25. The force necessary to separate the tape from the steel surface is measured in Newtons when the tape is detached from the plate with a constant rate of 100 mm per minute.

### 2.7. Peel Adhesion

The peel adhesion is the force required to remove a coated flexible pressure-sensitive adhesive sheet material from a test panel measured at a specific angle and rate of removal. For 180° peel measurements (AFERA 4001), the results depend on the face stock material. It is measured with a tensile testing machine, as described above.

### 2.8. Shear Strength

The shear strength is a value of the cohesive strength of an adhesive layer. It may be defined as a force required to remove an adhesive strip from a standard flat surface in a direction parallel to the surface to which it has been affixed. The strip has been bonded to the surface with a definite pressure. The procedure has been conducted according to AFERA 4012, at room temperature and at 70 °C.

## 3. Results

### 3.1. Characterization of the Synthesized Acrylic PSA

The acrylic pressure-sensitive adhesive prepared to possess the following features:

- amount of solid materials (polymer content) 50 wt %;
- viscosity 5.3 Pa·s;
- concentration of residual monomers < 0.1 wt %;
- weight average molecular weight M_W_ 41,000 Daltons;
- number average molecular weight Mn 135,000 Daltons;
- polydispersity Pd = M_W_/Mn 3.04.

### 3.2. Modification of Synthesized Acrylic PSA Using Testing Additionable Photoinitiators

**Figure 2:**
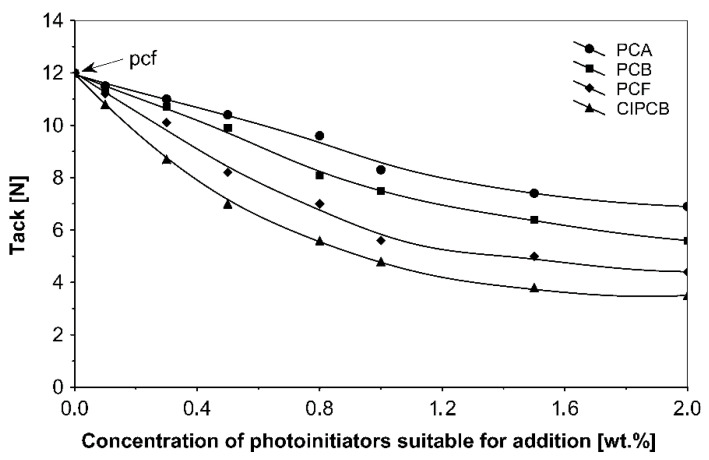
Tack of acrylic pressure-sensitive adhesives (PSAs) containing additionable photoinitiators after UV cross-linking.

**Figure 3:**
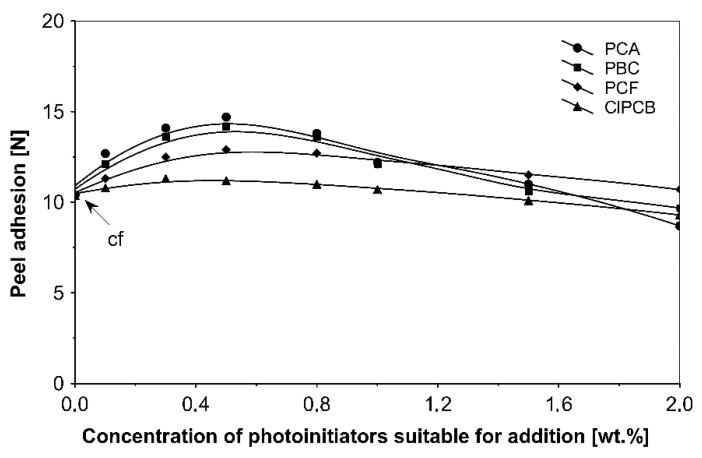
Peel adhesion of acrylic PSAs containing additionable photoinitiators after UV cross-linking.

**Figure 4:**
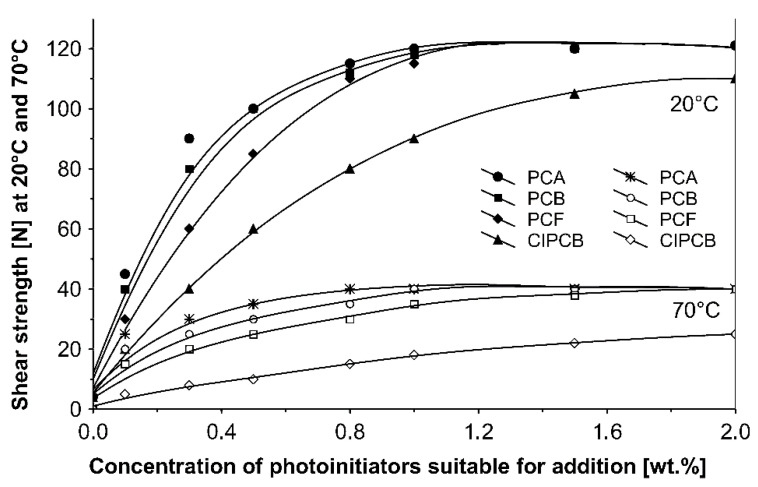
Shear strength of acrylic PSAs containing additionable photoinitiators after UV cross-linking.

The influence of tested additionable photoinitiators on tack (Figure 2), peel adhesion (Figure 3) and shear strength (Figure 4) of solvent-borne acrylic pressure-sensitive adhesives cross-linked with UV were investigated. The concentration of photoinitiator was: 0.1, 0.3, 0.5, 0.8, 1.0, 1.5 and 2.0 wt % according to polymer content.

Term pcf means partially cohesive failure, and cf means cohesive failure.

**Figure 5:**
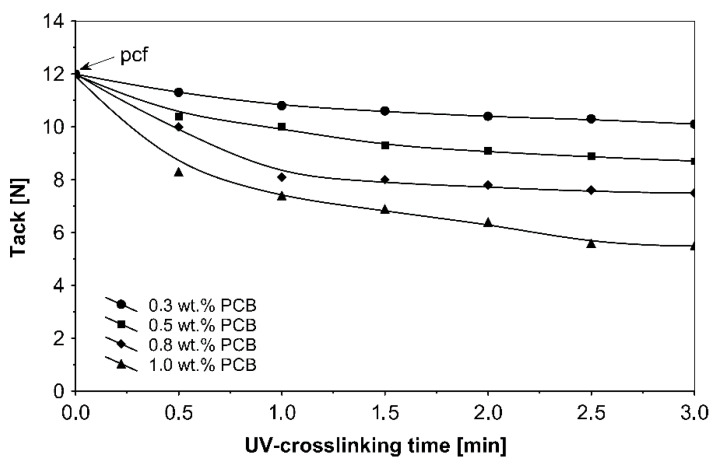
Tack of cross-linked acrylic PSAs containing various amounts of additionable photoinitiator versus UV-crosslinking time.

**Figure 6:**
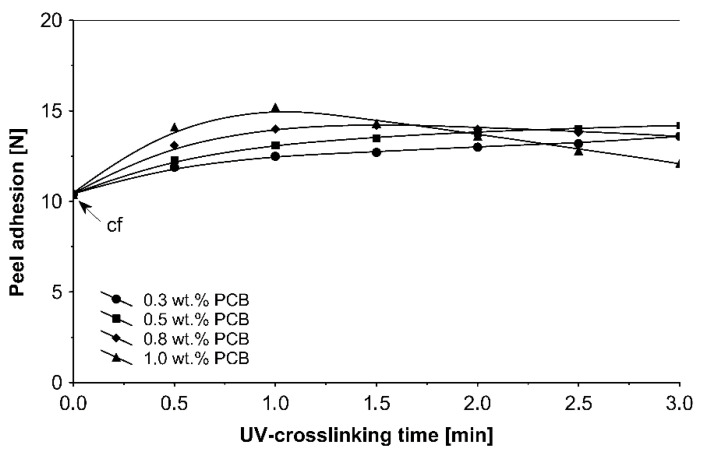
Peel adhesion of cross-linked acrylic PSAs containing various amounts of additionable photoinitiator versus UV-crosslinking time.

**Figure 7:**
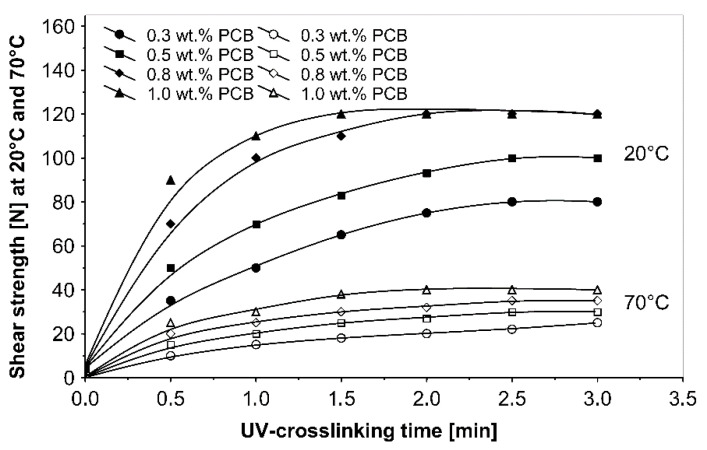
Shear strength of cross-linked acrylic PSAs containing various amounts of additionable photoinitiator versus UV-crosslinking time.

The UV-crosslinking time effect for the chosen photoinitiator 4-propyleneiminecarbonyl benzophenone in amounts ranging from 0.3 to 1.0 wt %, and for a UV radiation dose of 100 mJ/cm^2^, on adhesives properties of UV-crosslinked acrylic PSAs is shown in Figure 5, Figure 6 and Figure 7.

**Figure 8:**
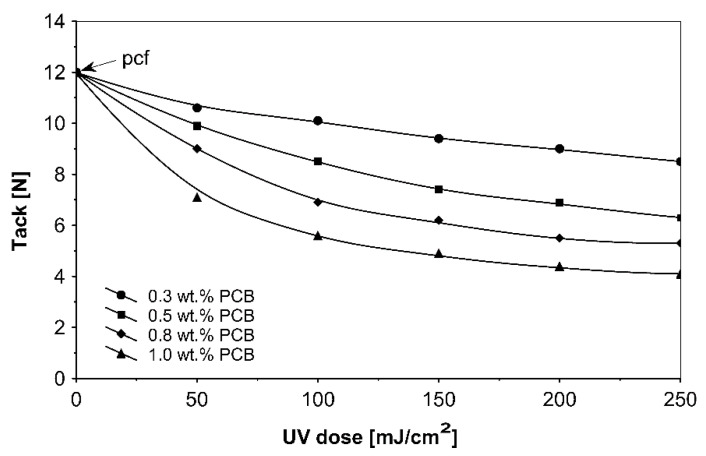
Tack of UV-crosslinked acrylic PSAs containing various amounts of additionable photoinitiator versus UV dose.

**Figure 9:**
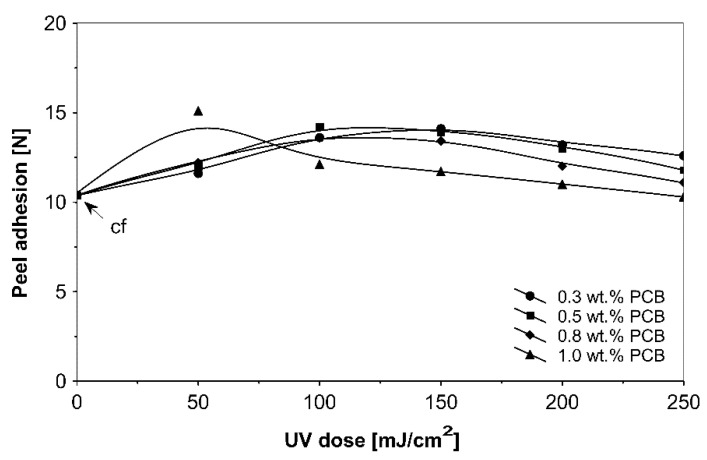
Peel adhesion of UV-crosslinked acrylic PSAs containing various amounts of additionable photoinitiator versus UV dose.

**Figure 10:**
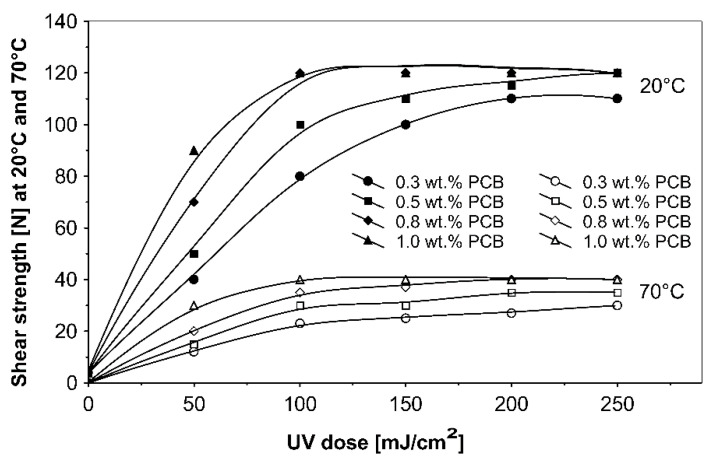
Shear strength of UV-crosslinked acrylic PSAs containing various amounts of additionable photoinitiator versus UV dose.

The ultraviolet cross-linking effect for the additionable photoinitiator PCB, in selected amounts between 0.3 and 1.0 wt % on adhesive properties of acrylic PSAs affected by UV doses ranging from 50 to 250 mJ/cm^2^ at 3 min irradiation time is shown in Figure 8, Figure 9 and Figure 10.

## 4. Discussion

It can be concluded from these experimental results that the all tested additionable photoinitiators had a negative influence on tack as a function of increasing the concentration of additionable photoinitiators (Figure 2), as a function of increasing the UV-crosslinking time (Figure 5) and as a function of UV dose (Figure 8). The uncrosslinked acrylic PSA layer, without an additionable photoinitiator, showed a high value of tack (initial adhesion) along with partially cohesive failure (pcf). After the addition of a small amount of additionable photoinitiators, the acrylic PSA under the UV lamp began to cross-link and the measured tack decreased. The PSA structure was then compact, peel adhesion increased, and in the course of the evaluation, a maximum of peel adhesion was observed. For PCA, PBC and PCF, the maximum was observed for 0.5 wt % photoinitiator and for ClPCB for ca. 0.3 wt % photoinitiator (Figure 3). A similar process was observed by UV cross-linking time by one-min UV exposure (Figure 6). The slightly differences are presented in Figure 9, where peel adhesion depended on an additionable photoinitiator PCB concentration of 1.0 wt %, showing maximum by a UV dose of 50 mJ/cm^2^. PSA layers containing 0.3, 0.5 and 0.8 wt % PCB showed maxima for UV dose between 100 and 150 mJ/cm^2^. Moreover, the highest shear strength levels, although at room temperature, especially at 70 °C, was attained by using additionable photoinitiator PCB containing in its chemical structure benzophenone group (Figure 4). With 1.0 wt % PCB were the maximal shear strength values of 120 N at room temperature and 40 N at 70 °C noted. For a 1.0 wt % PCB, we needed ca. 1.5 min (90 s) UV-crosslinking time (Figure 7) and about 100 mJ/cm^2^ UV dose to achieve the best shear strength of 120 N at room temperature and of 40 N at 70 °C.

**Figure 11:**
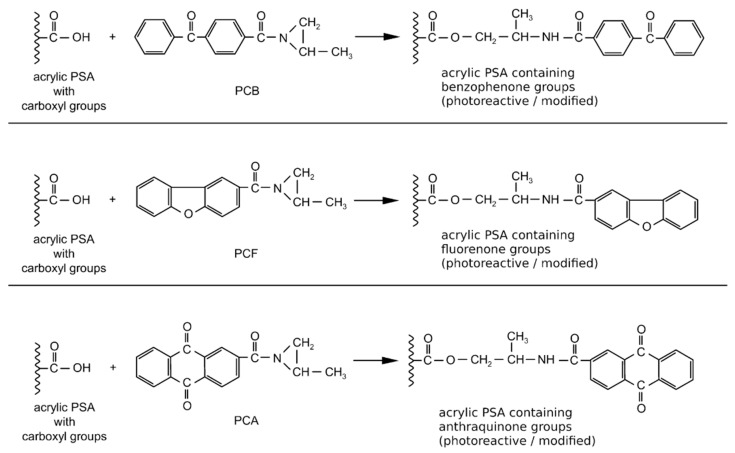
Reaction of additionable photoinitiator with carboxyl groups of acrylic PSA.

**Figure 12:**
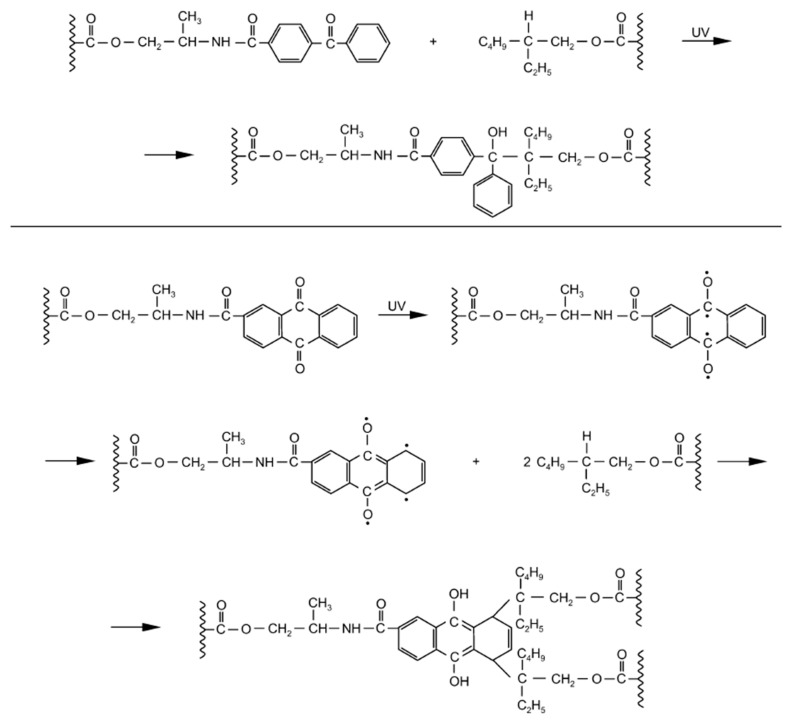
UV-initiated cross-linking of acrylic PSAs in the presence of additionable photoinitiators incorporated into the polymer backbone.

The incorporation of photoinitiator suitable for addition to the acrylic polymer chain containing carboxyl groups proceeded before the UV-crosslinking per the following reactions (Figure 11):

For example, two photoinitiators suitable for addition, those being 4-propyleneiminecarbonyl benzophenone (PCB) and 2-propyleneiminecarbonyl anthraquinone (PCA), were subjected to the UV-initiated cross-linking of acrylic pressure-sensitive adhesives possessing chemical structures described above (Figure 12):

The structure illustrated in Figure 12 seems to be very probable. The forming of this structure was not stereochemically hindered.

As it is seen from Figure 2, Figure 3 and Figure 4, the photoinitiators under study affected the properties of solvent-borne acrylic pressure-sensitive adhesives. For all PSAs containing additionable photoinitiators being studied, the decline of tack was achieved. By UV irradiation of PSA containing 4-chloro-4’-propyleneimine carbonyl benzophenone (ClPCB), a slight decrease of tack with an increasing amount of additionable photoinitiator was observed but compared with 2-propyleneiminecarbonyl anthraquinone (PCA), the tack effect was much less apparent.

Figure 3 demonstrates that using all tested additionable photoinitiators gave a peel adhesion optimum when their applied concentration was about 0.5 wt %. The highest peel adhesion values were observed for 2-propyleneiminecarbonyl anthraquinone (PCA) and 4-propyleneiminecarbonyl benzophenone (PCB).

The monitoring of the cohesion of UV-crosslinked acrylic PSAs was undertaken using shear strength, measuring at 20 °C and 70 °C in this study (Figure 4). It was found that the shear strength progressed even if more photoinitiator was added to the UV cross-linkable acrylic adhesive. The acrylic PSA containing (PCA) reacted most rapidly and (ClPCB) was worst, which can be explained by the chlorine atom factor of the substituents. For about 0.8 wt % (PCA) and (PCB), and for 1.0 wt % (PCF), after UV cross-linking, the maximum shear strength at room temperature of 120 N was reached. The highest shear strength at 70 °C of 40 N was achieved for 0.8 wt % (PCA), for 1.0 wt % (PCB) and for 2.0 wt % (PCF). Shear strength is evaluated in [N/2.5 cm × 2.5 cm] also in [N/6.25 cm²].

It was found that the acrylic PSAs containing 4-propyleneiminecarbonyl benzophenone (PCB) exhibit optimal balance of shear strength, peel adhesion and tack after UV radiation. In acrylic PSA, the photoreactivity of 4-propyleneiminecarbonyl benzophenone (PCB) was inferior to other tested additionable photoinitiators.

Taking into account the results presented in Figure 5, Figure 6 and Figure 7, one can conclude that the UV-crosslinking time was found to have a distinct effect on the peel adhesion of the ultraviolet cross-linked acrylic pressure-sensitive adhesives (Figure 6). For 1.0 wt % (PCB), a peel adhesion maximum of 15.2 N at 60 s UV exposure was noticed. For 0.8 wt % (PCB), the maximum value of peel adhesion was noticed at 1.5 min UV exposure. For 0.3 wt % (PCB) and for 0.5 wt % (PCB), the peel adhesion increases with an increase up to 3 min of UV-crosslinking time.

High curing efficiency is one of the major requirements for photoinitiators used in UV cross-linkable pressure-sensitive adhesives. Figure 7 shows the shear strength at 20 °C and 70 °C versus various 4-propyleneiminecarbonyl benzophenone (PCB) amounts. With 1.0 wt % and 0.8 wt % (PCB), significantly high cohesion values were stated, after 90 s UV exposure 120 N for 1.0 wt % (PCB) and after 2 min UV curing the same shear strength value for 0.8 wt % (PCB). The highest cohesion value of 40 N at 70 °C was only possible by the use of 1.0 wt % (PCB).

As it is presented above, the ultraviolet cross-linking effect depends on UV doses (Figure 8, Figure 9 and Figure 10). Therefore, the tack behavior strongly is affected by the emitted UV dose and the (PCB) content. It was also shown that the UV dose is also an important parameter. By increasing the UV dose, the tack of UV-crosslinked acrylic PSAs decreases rapidly.

Moreover, an acrylic PSA containing 1.0 wt % (PCB) gives a peel adhesion maximum of 15.1 N at 50 mJ/cm^2^ UV dose. With the increasing dose of the UV lamp, a decrease in peel adhesion could be observed. By applying 0.3 wt % (PCB), a peel adhesion maximum of 14.1 N was noticed for 150 mJ/cm^2^ UV dose.

Basing on the results shown in Figure 10, the influence of the additionable photoinitiator (PCB) in different concentrations on the shear strength profiles at 20 °C and 70 °C, of UV-crosslinked acrylic pressure-sensitive adhesives exposed to intense UV radiation the following cohesion ranking for all UV doses at 20 °C and 70 °C was obtained: 1.0 wt % (PCB) > 0.8 wt % (PCB) > 0.5 wt % (PCB) > 0.3 wt % (PCB). The increase of the UV dose influences the shear strength positively. With 0.3 wt % (PCB), the highest cohesion values at 20 °C and 70 °C were not achieved. By increasing the curing level and the (PCB) content, the cohesion of UV-crosslinked acrylic PSAs gets stronger.

## 5. Conclusions

It was shown here an achieving innovative, and competitive solutions tailored to the customer’s needs is possible through the formulation, application, degree of cross-linking or a combination of main parameters such as adhesion, peel adhesion and shear strength.

Photoreactive solvent-borne acrylic pressure-sensitive adhesives (PSA) are not commercially available on the market. Without admittance of UV radiation, they are very stable and each time suitable for industrial application for the production of pressure-sensitive materials. New additional photoinitiators for UV curable acrylic PSA formulations can help to increase pot-life and reduce the viscosity of UV cross-linkable PSAs.

## Figures and Tables

**Table 1:** The photoinitiators under study.

**Photoinitiato**r	**Chemical Formula**	**Chemical Name**
PCB	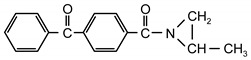	4-propyleneiminecarbonyl benzophenone
PCF	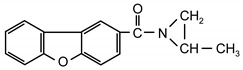	2-propyleneiminecarbonyl-dibenzofurane
ClPCB	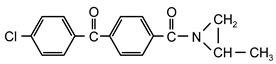	4-chloro-4’-propyleneiminecarbonyl benzophenone
PCA	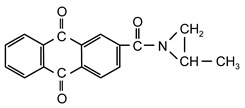	2-propyleneiminecarbonyl anthraquinone
